# Supplementary material for: Graphene-integrated mesh electronics with converged multifunctionality for tracking multimodal excitation-contraction dynamics in cardiac microtissues
Source: Nat Commun. 2024 Mar 14;15:2321. doi: 10.1038/s41467-024-46636-7 (PMC10940632; doi:10.1038/s41467-024-46636-7)
Supplement: Supplementary file 1 — Supplementary Information [file 41467_2024_46636_MOESM1_ESM.pdf]

Supplementary Information for

Graphene-Integrated Mesh Electronics with Converged Multifunctionality for Tracking Multimodal Excitation-Contraction Dynamics in Cardiac Microtissues

Hongyan Gao,[1] Zhien Wang,[2] Feiyu Yang,[3] Xiaoyu Wang, [1] Siqi Wang, [1] Quan Zhang, [1] Xiaomeng Liu,[1] Yubing Sun,[3,4,5] Jing Kong, [2] Jun Yao*[1,4,5]

---

[1]Department of Electrical and Computer Engineering, University of Massachusetts, Amherst, 01003, USA.
[2]Department of Electrical Engineering and Computer Science, Massachusetts Institute of Technology, Cambridge, 02139,USA.
[3]Department of Mechanical and Industrial Engineering, University of Massachusetts, Amherst, 01003, USA.
[4]Institute for Applied Life Sciences, University of Massachusetts, Amherst, 01003, USA.
[5]Department of Biomedical Engineering, University of Massachusetts, Amherst, 01003, USA.

* Corresponding author. E-mail: juny@umass.edu (J.Y.)

This file includes:

Supplementary Figures S1 – S32

Supplementary References

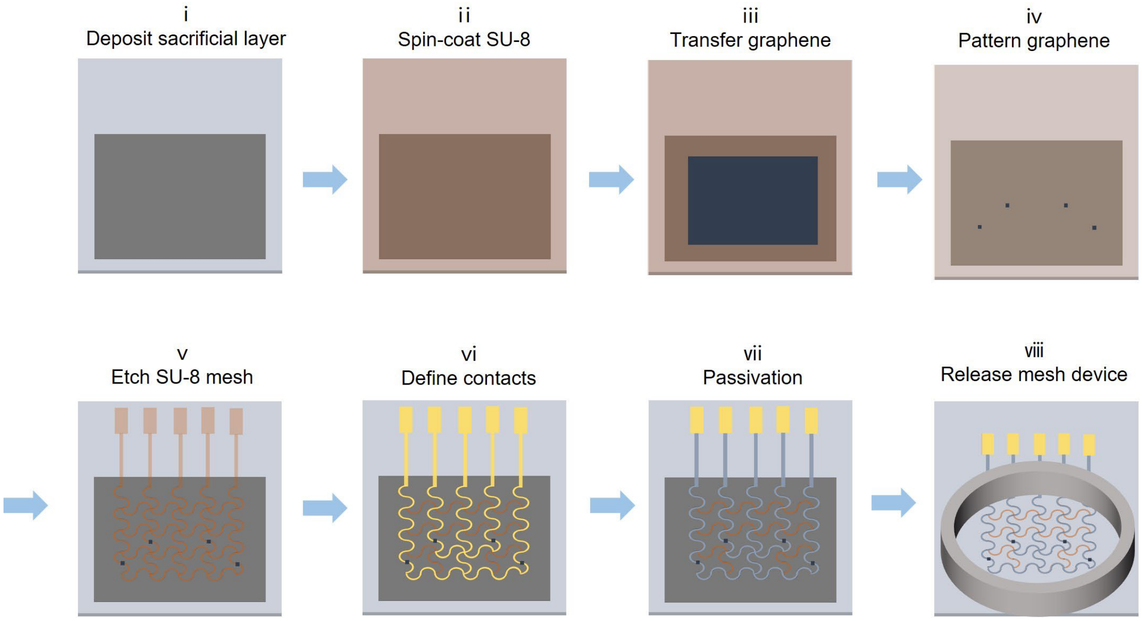

Fig. S1. Fabrication flow of the integrated mesh system. (**i**) A Si wafer (covered with 600 nm SiO_2, Nova Electronics Materials) or glass wafer (Soda lime glass, University Wafer) was precleaned by acetone, isopropyl alcohol (IPA), and deionized (DI) water. Photolithography and electron-beam evaporation were used to define a selected area of Ge layer (120 nm thick) as the sacrificial layer. (**ii**) A layer of SU-8 (2000.5, Kayaku Advanced Materials) was spin-coated on the substrate and hard-baked (180 ^{o}C for 30 min). (**iii**) The polymethyl methacrylate (PMMA)/graphene layer (*e.g.*, obtained by etching away the copper growth substrate in CE-100) was thoroughly cleaned (*e.g.*, rinse with DI water for 10 times) and transferred on to the SU-8 layer, which was pre-treated with oxygen plasma (50 W, 20 sccm O_2, 30 s) for increasing surface hydrophilicity. The substrate was then baked to improve adhesion before the PMMA layer was removed in acetone (30 min). More details about the transfer process can be found in Fig. S2. (**iv**) Photoresists (PMGI SF6 and S1805) were spin-coated on the substrate and photolithography was carried out to pattern graphene transistor array. Graphene in the unprotected area was by etched away by oxygen plasma (50 W, 50 sccm O_2, 1 min), and the remaining photoresist was removed by PG Remover. (**v**) Double-layer photoresists of PMGI SF6 and S1813 were spin-coated on the substrate and photolithography was carried out to define the mesh pattern. The unprotected SU-8 area was etched away by oxygen plasma (RF=100 W, ICP=1000 W, 50 sccm O_2, 30 s), and remaining photoresists were removed by PG Remover. (**vi**) Photolithography and metal deposition were used to define interconnects (Cr/Pd/Au/Pd, 3/15/40/5 nm). (**vii**) The interconnects were further passivated by sputtering a layer of Si_3N_4 (~70 nm) to prevent current leakage in solution environment. (**viii**) The fabricated mesh system was released from the substrate by etching away the Ge layer in 1% H_2O_2 solution (30 min) and rinsed clean in DI water before cell culture.

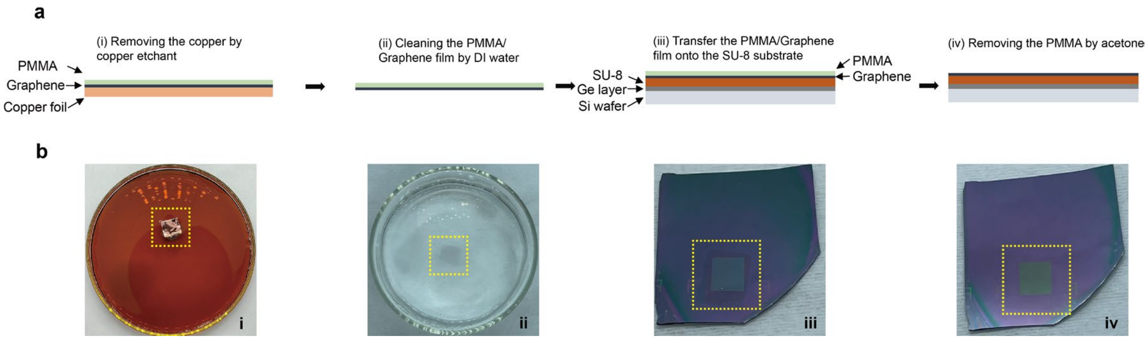

Fig. S2. Transfer of graphene. **a**, Schematics of the transfer process. (i) Monolayer graphene grown on the backside of a Cu foil was spin-coated with a PMMA layer (~ 400 nm) and baked at 80ºC for 5 min. The unprotected frontside graphene was etched away by oxygen plasma (50 W, 2 min, 50 sccm O_2). The sample was then floated on the Cu etchant (CE-100, Transene, Inc.) for 30 min to remove the Cu foil. (ii) The released PMMA/graphene film was rinsed with DI water for 10 times to remove Cu-etchant residue and stayed floating on DI water. (iii) The substrate coated with a SU-8 layer (hard-baked at 180 ºC for 30 min) was treated by oxygen plasma (50 W, 20 sccm O_2, 30 s) to make the surface hydrophilic. The substrate was then immersed in the DI water to pick up the PMMA/graphene film, and then baked at 100ºC for 5 min to dry and improve the adhesion between graphene and SU-8. (iv) The PMMA film was removed by immersing the sample in acetone for 30 min. Subsequent fabrication processes as described in Fig. S1 were carried out for mesh fabrication. **b**, Actual steps of (i) removing the Cu foil by Cu etchant, (ii) cleaning the PMMA/graphene film by DI water, (iii) transferring the PMMA/graphene film onto the SU-8 substrate, and (iv) removing the PMMA film by acetone.

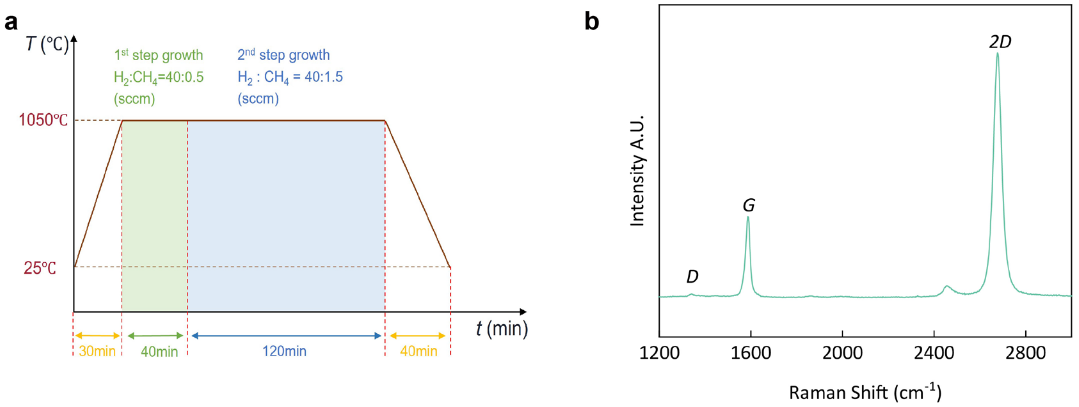

Fig. S3. Graphene synthesis and Raman characterization. **a**, Synthesis flow of monolayer graphene on copper foil. **b**, Raman spectrum of pristine graphene before device fabrication. The small *D* peak (~1340 cm^{-1}) indicates low defect in the graphene. The ratio between the *2D* peak (~2675 cm^{-1}) and *G* peak (~1584 cm^{-1}) was ~2.9, suggesting single layer of graphene.[1] 5 spots were measured on each sample and the Raman signals from these different spots showed consistent results.

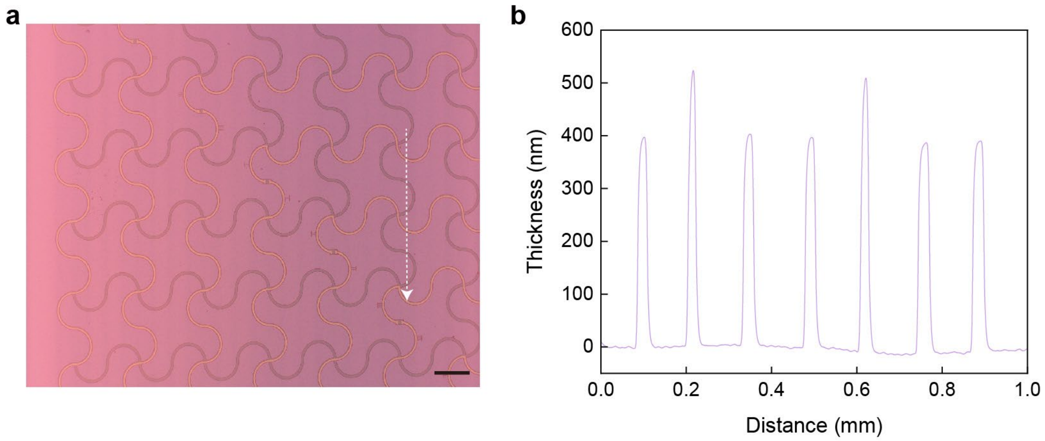

Fig. S4. Mesh thickness characterization. **a**, Optical image of a fabricated mesh system before releasing from the substrate. Scale bar, 200 µm. The dash arrow indicates the scanning direction for thickness measurement (Dektak Profilometer, Bruker). **b**, The thickness profile of the mesh ribbon (~400 nm) and mesh ribbon containing interconnect and passivation layers (~530 nm).

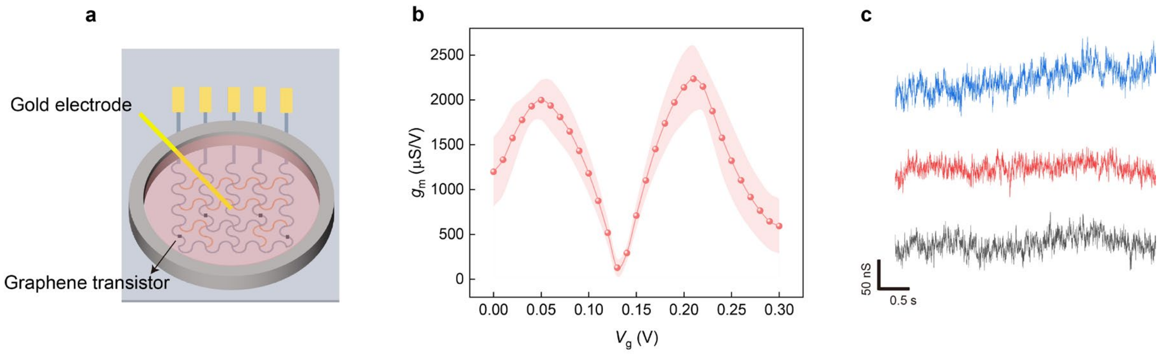

Fig. S5. Noise characterization in graphene devices. **a**. Schematic setup for measuring water-gate effect in graphene transistors. The water-gate voltage (V_g) was applied to the Dulbecco's phosphate-buffered saline (DPBS) solution by a gold-wire electrode. **b**, Average transconductance (g_m) of graphene field effect transistors at different water-gate voltage, showing a peak value ~2.2 ± 0.4 mS/V. **c**, Conductance fluctuations in the graphene transistors (at V_g = 0.21 V), showing noise level ~ 20-40 nS.

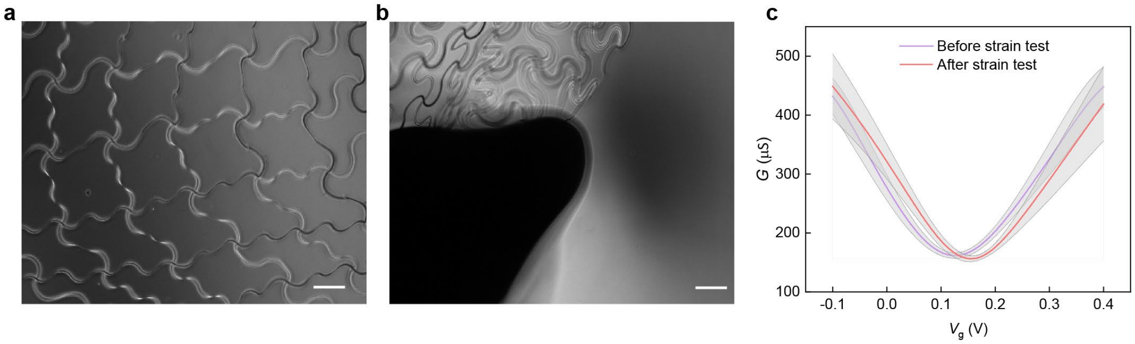

Fig. S6. Mesh system under mechanical deformation. Optical images of a released mesh (in water) being (**a**) stretched (~30% biaxial strain) and (**b**) folded (180°) by a tweezer. Scale bars, 200 μm. **c**, Conductance (G) *vs.* water-gate voltage (V_g) recorded from 8 graphene transistor devices integrated on the mesh immersed in DPBS solution before (purple) and after (red) 100 cycles of stretching and folding. The lines and shadow represent mean values and the standard deviation, respectively. The water-gate voltage was applied to the DPBS solution by a gold-wire electrode as illustrated in Fig. S5a.

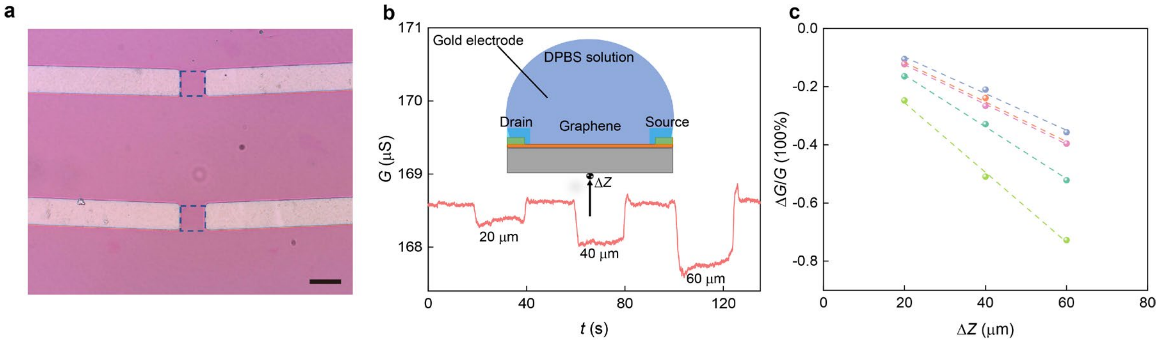

Fig. S7. Piezoresistive effect in graphene transistors. **a**, Optical image of planar graphene transistors (dash box, size ~20×20 μm^2) on a Si substrate. Scale bar, 20 μm. **b**, Representative conductance (G) change with respect to the increase of vertical displacement (ΔZ) in the substrate. The inset shows the schematic setup for bending the substrate. Specifically, graphene transistors were fabricated at the central region of a rectangular Si substrate (4 cm × 4 cm). The lateral edges of the substrate were mechanically fixed. A sapphire bead (3 mm diameter) was placed beneath the substrate center and displaced by a micrometer in the vertical direction to bend the substrate. A fixed water-gate voltage (V_g) was applied to the by a gold wire through a droplet of DPBS solution. **c**, Relative conductance change ($\Delta G/G$) with respect to ΔZ from 5 graphene transistors. The average slope was −(8.17 ± 2.36) ×10^{-3} μm^{-1}. The strain ε in the graphene device can be estimated as $\varepsilon = \tau/2R$,[2] where τ is the substrate thickness (400 μm) and R is the radius of the bending curvature. The estimated strains for vertical displacements (ΔZ) of 20 μm, 40 μm and 60 μm are ~2×10^{-5}, 4×10^{-5} and 6×10^{-5}, respectively. The average gauge factor is $g = \frac{\left(\frac{\Delta R}{R}\right)}{\Delta \varepsilon} = -\frac{\left(\frac{\Delta G}{G}\right)}{\Delta \varepsilon} = 81.7 \pm 23.6$.

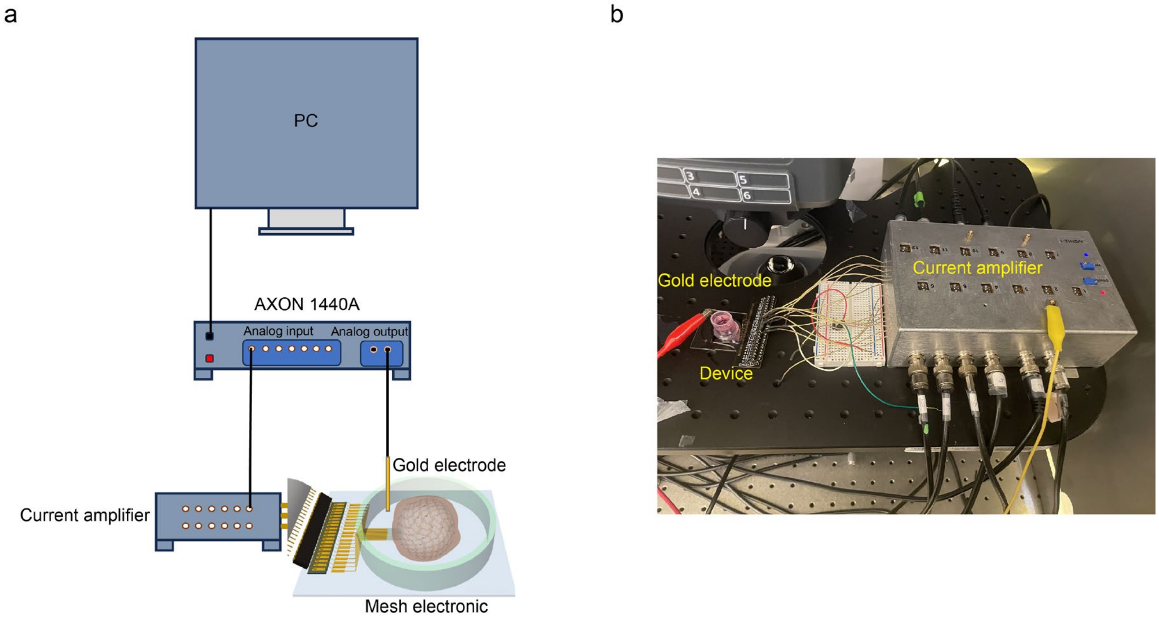

Fig. S8. Experimental setup for recording mesh-innervated CMTs. **a**, The drains of the graphene transistor devices were connected to a home-built (multichannel) current amplifier. The amplified signals were connected to an analog-to-digital converter (digidata 1440A). The converted data was acquired by a computerized software (pClamp 10.7). During the recording, a fixed water-gate voltage was applied to the cell culture medium by a gold electrode to provide a stable global reference. **b**, Optical image of the actual setup.

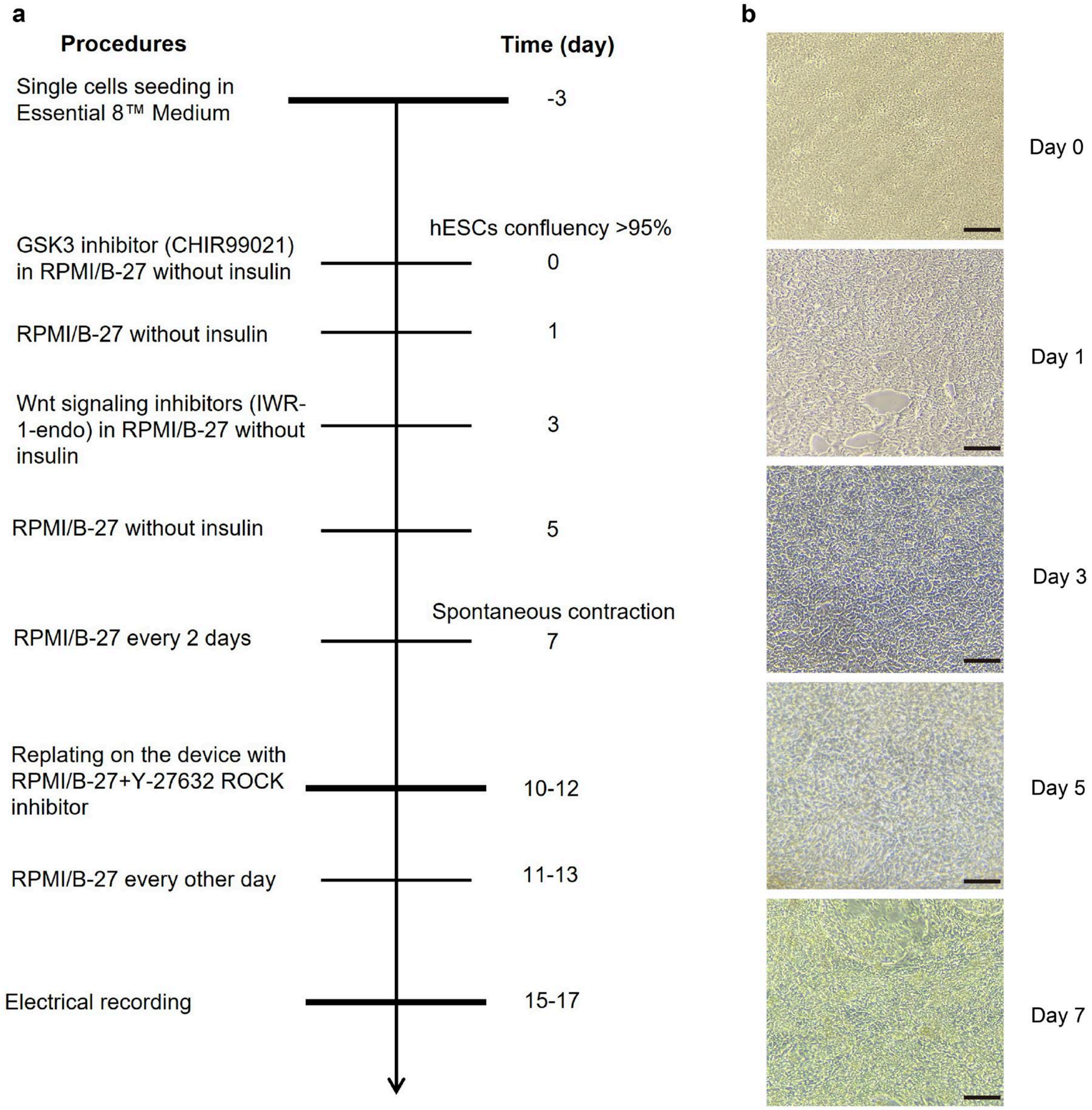

Fig. S9. Timeline for mesh-CMT integration. a, Timeline for cell differentiation, integration of mesh electronics, and electrical recording. The differentiated cardiomyocytes were transferred onto the mesh during days 10 to 12. The mesh was gradually embedded into the CMT by tissue growth and folding process (Fig. 2b in main text). The electrical recording was started on days 15 to 17. **b**, Brightfield optical images showing increasing cell densities during the differentiation. Scale bar, 80 μm.

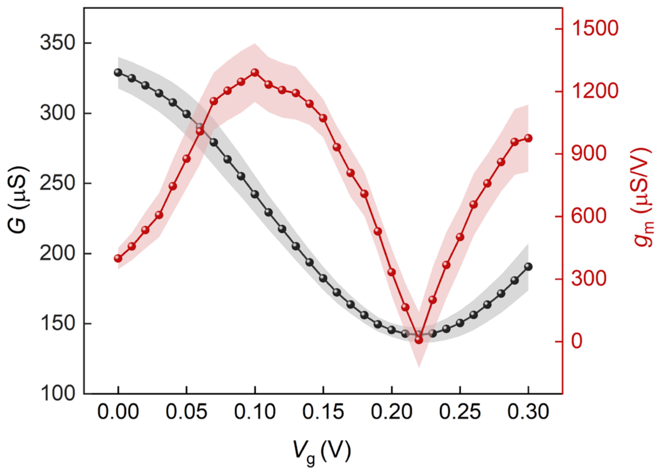

Fig. S10. Calibrating sensing signals in Fig. 2. Conductance (G) and transconductance (g_m) with respect to the water-gate voltage (V_g) in mesh-integrated graphene transistors embedded in a CMT. The lines and shadow represent mean values and the standard deviation, respectively. The recordings (Fig. 2 main paper) were performed with fixed V_g=0.1V, corresponding to a transconductance ~1.3 mS/V. The recorded action-potential signals (conductance) correspond to a calibrated voltage range of 70 to 200 μV. The water-gate voltage was applied to the cell culture medium by a gold-wire electrode as illustrated in Fig. S8.

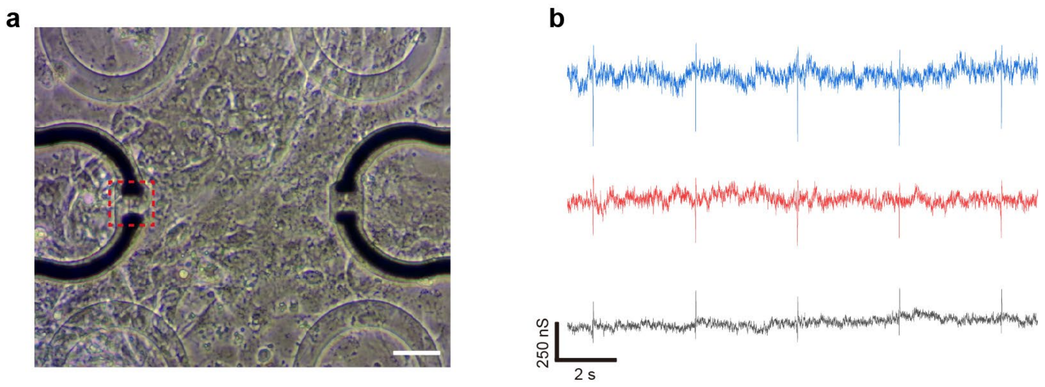

Fig. S11. Electrical recordings from a non-released mesh. **a**, Optical image of a planar mesh system cultured with a layer of cardiomyocytes. The dash box indicates the graphene transistor. Black wires are the metal interconnects. Scale bar, 40 μm. **b**, Electrical recordings showing periodic action-potential signals, which are consistent with previous planar graphene transistors for recording action potentials.[3] No broad (mechanical) peak was observed.

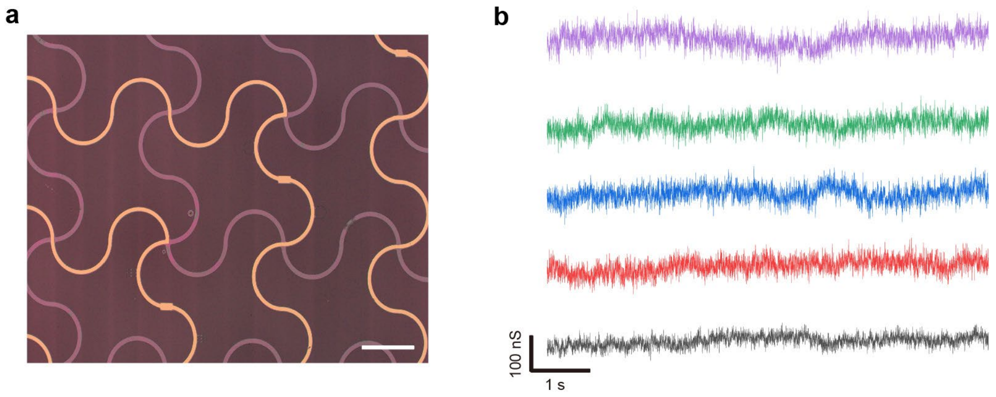

Fig. S12. Electrical recordings from a mesh (with only metal interconnects). **a**, Optical image of the mesh fabricated with only metal interconnects (without graphene transistors). Scale bar, 200 µm. **b**, Five-channel recordings from the mesh innervated in a CMT. No electrical or mechanical signal peak was observed.

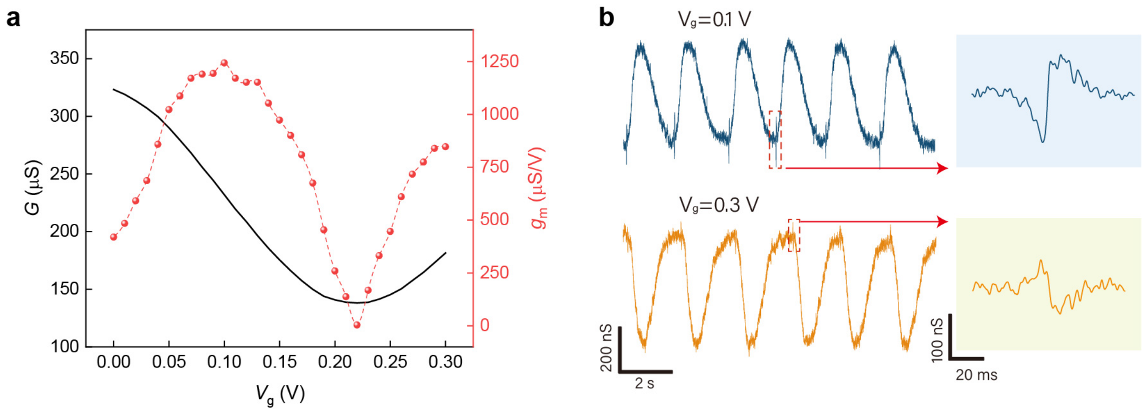

Fig. S13. Sensing signals in different transport regions. **a**, Conductance (G) and transconductance (g_m) with respect to the water gate (V_g) from the 5th graphene transistor (from top to bottom, Fig.2 in main paper), showing a *p*- to *n*-type transition at $V_g \sim 0.22$ V. **b**, Bioelectrical sensing signals recorded by biasing (V_g=0.1 V, top) the graphene device in the *p*-type transport region and *n*-type region (V_g=0.3 V, bottom) showed that the signs of both the mechanical signal and action-potential signal (zoom-in panel to the right) flipped. The sign flipping in the action-potential signal is expected from the *field*-effect mechanism, whereas the sign flipping in the mechanical signal is attributed to the sign change in the gauge factor (see Fig. S14) as also reported previously.[4] The water-gate voltage was applied to the cell culture medium by a gold-wire electrode as illustrated in Fig. S8.

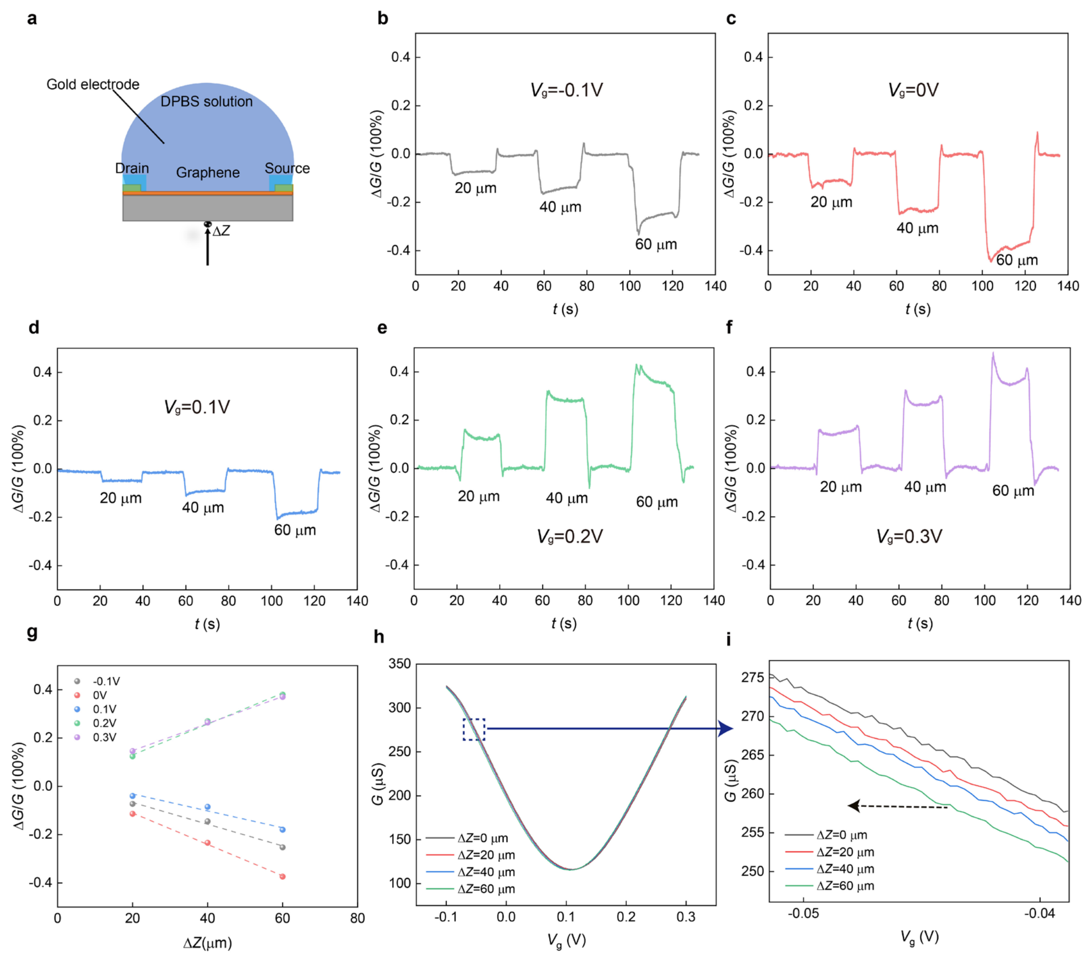

Fig. S14. Gate-dependent piezoresistive effect in graphene transistors. **a,** Schematic setup for characterizing gate-dependent piezoresistive effect in graphene transistors. The water-gate voltage (V_g) was applied to the DPBS solution by a gold-wire electrode. The bending strain in the graphene transistor was applied through a vertical displacement (ΔZ from 20 μm to 60 μm) in the substrate at different water gate (V_g) of -0.1 to 0.3 V. **b-f**, Relative conductance change ($\Delta G/G$) in a graphene transistor with respect to bending train. **g**, Summary of the piezoresistive effect recorded from (a-e), showing an opposite trend between $V_g \leq 0.1$ and ≥ 0.2V. The opposite trend suggests a reverse of sign in the gauge factor. **h**, Strain effect on the transport in the graphene transistor, showing (right panel **i**) a left shift in the transport curve with increasing strain (applied by the vertical displacement ΔZ in the substrate). This means that the increasing strain reduces the conductance in the *p*-type region but increases the conductance in *n*-type region, showing an opposite piezoresistive effect in the two regions consistent with measurements in (f). This phenomenon is also consistent with the previous test at low temperature and the cause was attributed to a strain-induced scalar potential.[5]

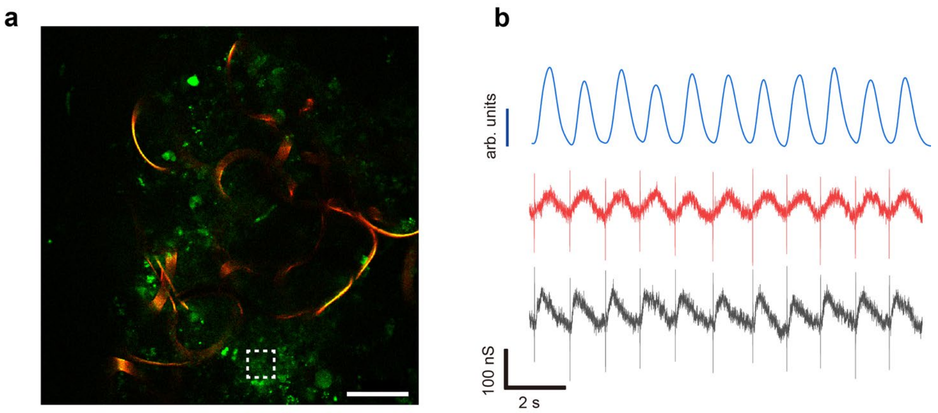

Fig. S15. Ca^{2+}-transient imaging and electrical recordings. **a**, Optical Ca^{2+} signals from a mesh-innervated CMT. The green (Fluo-8 AM) and red colors indicate the Ca^{2+} transient and mesh, respectively. The dash box indicates the region of interest (ROI) for Ca^{2+}-transient analysis. Scale bar, 100 μm. **b**, Evolution of the Ca^{2+} intensity (blue) from the ROI and electrical signals (black and red) recorded by two graphene devices. The Ca^{2+}-transient signal was recorded at 7.5 fps (~133 ms per frame). Imaging details can be found in the *Methods*. Note that the strong action-potential amplitudes in the electrical recordings suggest a robust device-cell interface. The relatively low amplitudes in the mechanical signals, which still fall into the typical range (Fig. 2d main text), may be attributed to geometric effect (e.g., a parallel alignment between the device plane and contractile direction can minimize the strain effect).

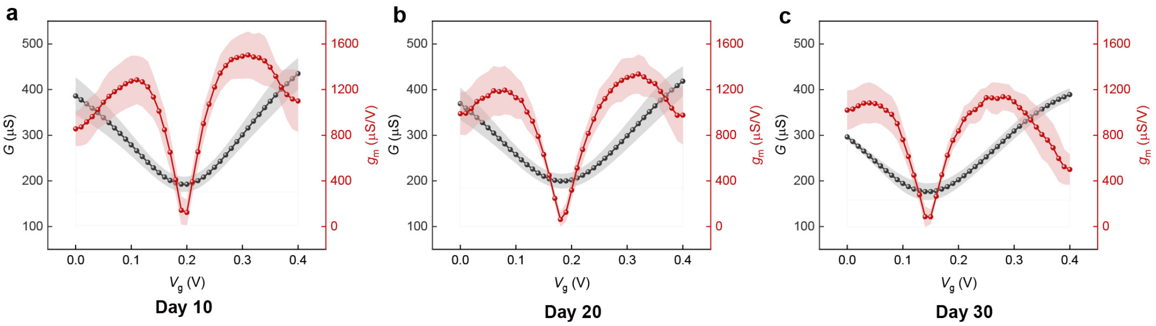

Fig. S16. Electrical characterization of graphene devices embedded in a CMT. **a-c**, Conductance (G) and transconductance (g_m) in the graphene devices with respect to the water gate (V_g) at day 10, day 20, and day 30 after seeding cell on mesh device, respectively. The lines and shadow represent mean values and the standard deviation (n=5 independent transistors). The peak transconductance (in the p-type region) changed from ~1.28 mS/V to 1.08 mS/V and the Dirac point shifted from ~0.2 V to 0.14 V. Similar shift was observed in previous graphene transistors used for bioelectronic chronic recording.[6] The water-gate voltage was applied to the cell culture medium by a gold-wire electrode as illustrated in Fig. S8.

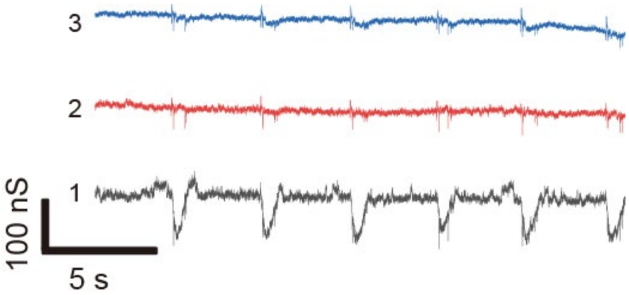

Fig. S17. Electrical recordings of the CMT at day 16 of differentiation (day 5 after seeding). The numbers 1, 2, 3 represent signals recorded from three spatially distributed sensors. Device 1 captured the early development of obvious mechanical contraction; device 3 captured the beginning development of mechanical contraction; and device 2 showed a delayed initiation of mechanical signal. These results show that cell development can have regional differentiation (e.g., in timeline) and can be captured by the spatially distributed sensors.

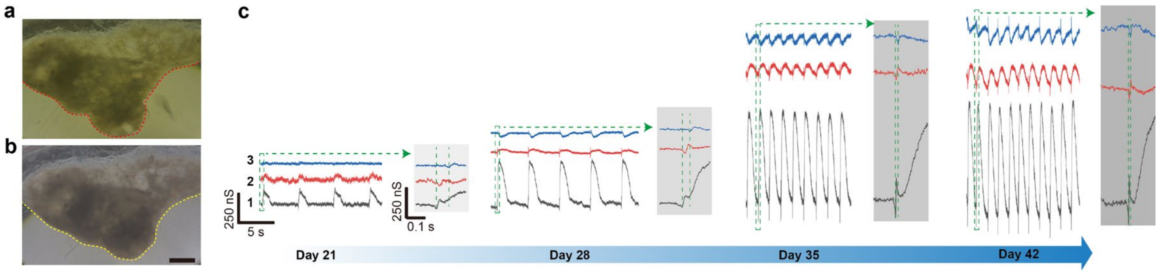

Fig. S18. Temporal signal delay between different graphene devices. a-b, Optical images of the mesh-innervated CMT after 21 days and 41 days of differentiation (days 10 and 30 of cell seeding). Scale bar, 200 µm. The red and yellow dashed lines delineate the boundary of CMT, showing minimal morphological change in the CMT during the continuous development. This also suggests that the relative spatial distance (*S*) between two embedded graphene devices does not change over time. **c**, Representative recordings from three devices at days 21, 28, 35, 42 of differentiation (days 10, 17, 24, 31 of cell seeding). The right panel shows zoom-in signals from the dash box in each recording. The (temporal) distance between each pair of dash lines defines the time delay (t) in action potentials recorded by the two graphene devices. Since the electrical conduction velocity can be calculated by $v = S/t$, the decreasing delay t suggests an increasing electrical conduction velocity in the tissue. Channels 1, 2 and 3 correspond to the three channels in Fig. S17.

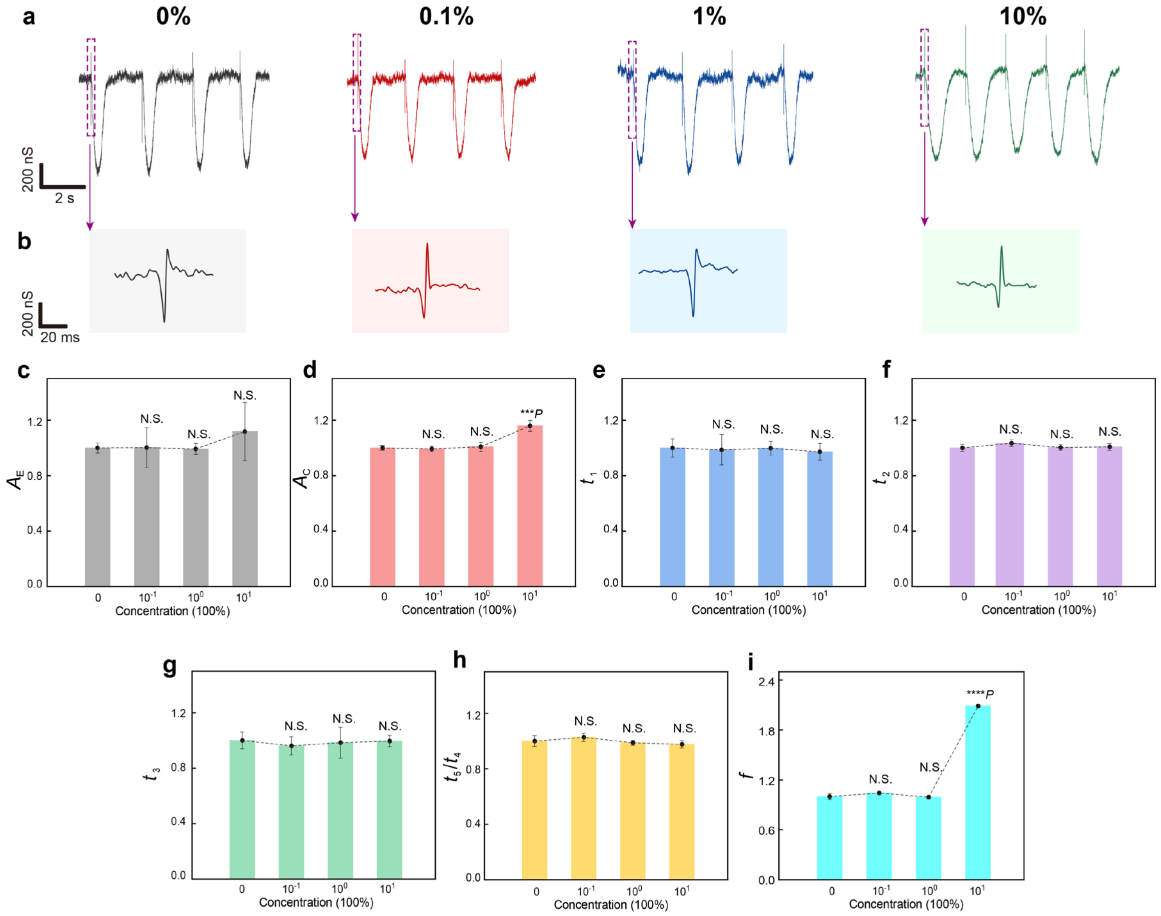

Fig. S19. Dose-dependent dimethyl sulfoxide (DMSO) effect on CMT. a, Representative recordings from the same graphene device embedded in the CMT treated with different DMSO concentrations. **b**, Zoom-in action-potential signals. **c-i**. Statistical summary of the extracted parameters (defined in Fig. 3a in main paper) at different DMSO concentrations (n=4 independent recording signals). All values are normalized to the initial value before DMSO treatment. The results suggest that the physiological response in the CMT remained stable when it was treated with DMSO concentration < 1%. All the drug tests performed in this study used DMSO (as a carrier solution) concentration < 1%. Data in (c-i) are presented as mean values ± SD. ***P < 0.001, ****P < 0.0001, N.S. not significant, using one-way ANOVA with the concentration 0% group as control.

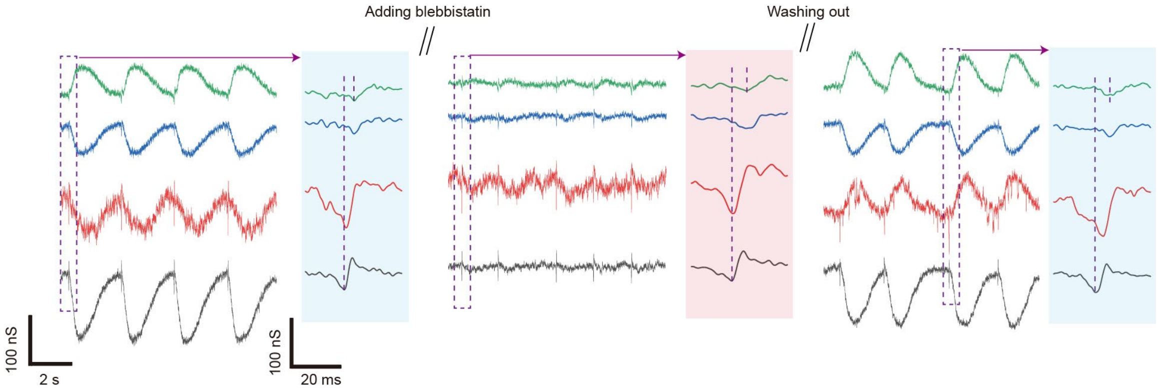

Fig. S20. Recordings of Blebbistatin effect. Recordings from the mesh-innervated CMT before adding Blebbistatin (left), ~140 s after adding Blebbistatin (middle), and washing out Blebbistatin (right). The right panel shows the zoom-in action-potential signals in each recording to the left (boxed region).

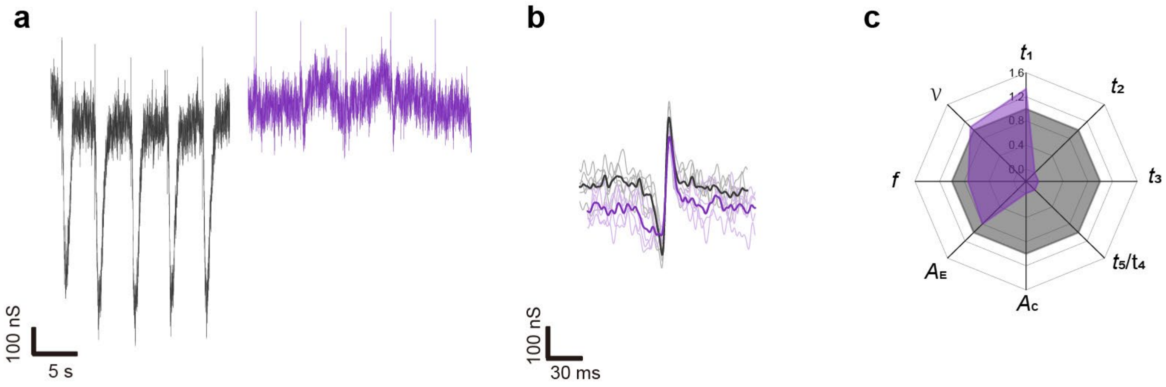

Fig. S21. Recordings of Verapamil effect on CMT. **a**, Electrical recordings from a CMT before (black) and after (purple) adding 1 μM Verapamil. **b**, Superimposed action-potential signals before and after adding Verapamil, with the black and purple curves representing the mean waveforms. **c**, Radar map using the extracted parameters (defined in Fig. 3a in the main paper). The grey and purple patterns represent the normalized values before and after Verapamil treatment, respectively. The radar map can be readily differentiated from the Blebbistatin effect (Fig. 4c in main paper).

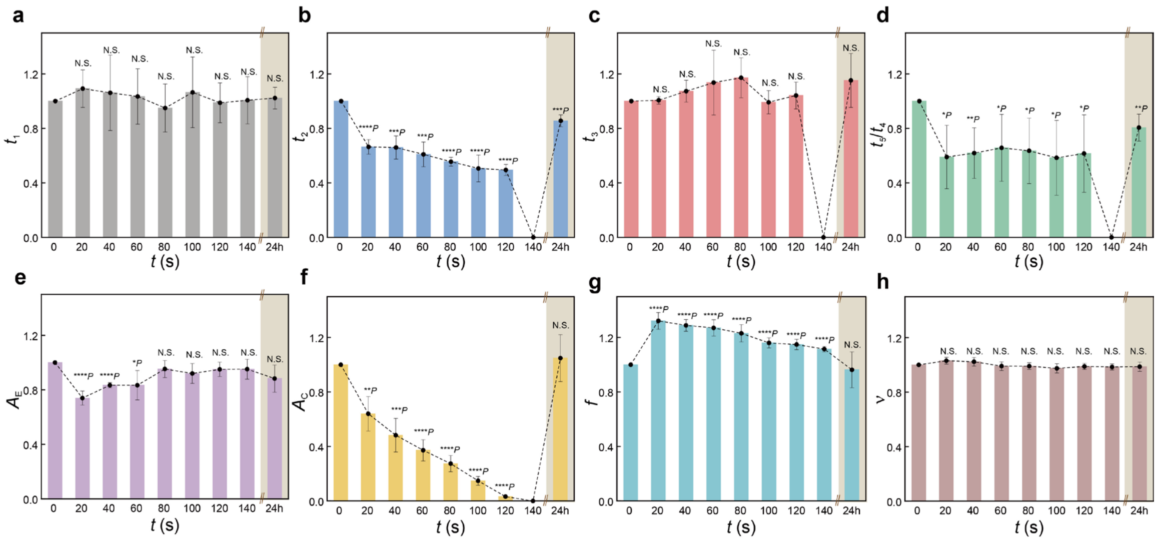

Fig. S22. Signal evolution from Blebbistatin-treated CMT. a-h, Statistical summary of extracted features (defined in Fig. 3a in the main paper) from the recording signals (*n*=4 independent devices) before, 140 s after adding the drug, and 24 h after washing it out (shadow areas). All values are normalized to the initial value before Blebbistatin treatment. The results suggest that Blebbistatin mainly affected the mechanical function of CMT but had negligible effect on the electrical activity. The recordings show that the CMT recovered 24 h after washing out the drug, demonstrating that the mesh system could closely track drug effect at different stages. Data are presented as mean values ± SD. *$P < 0.05$, **$P < 0.01$, ***$P < 0.001$, ****$P < 0.0001$, N.S. not significant, using one-way ANOVA with the t=0 s group as control.

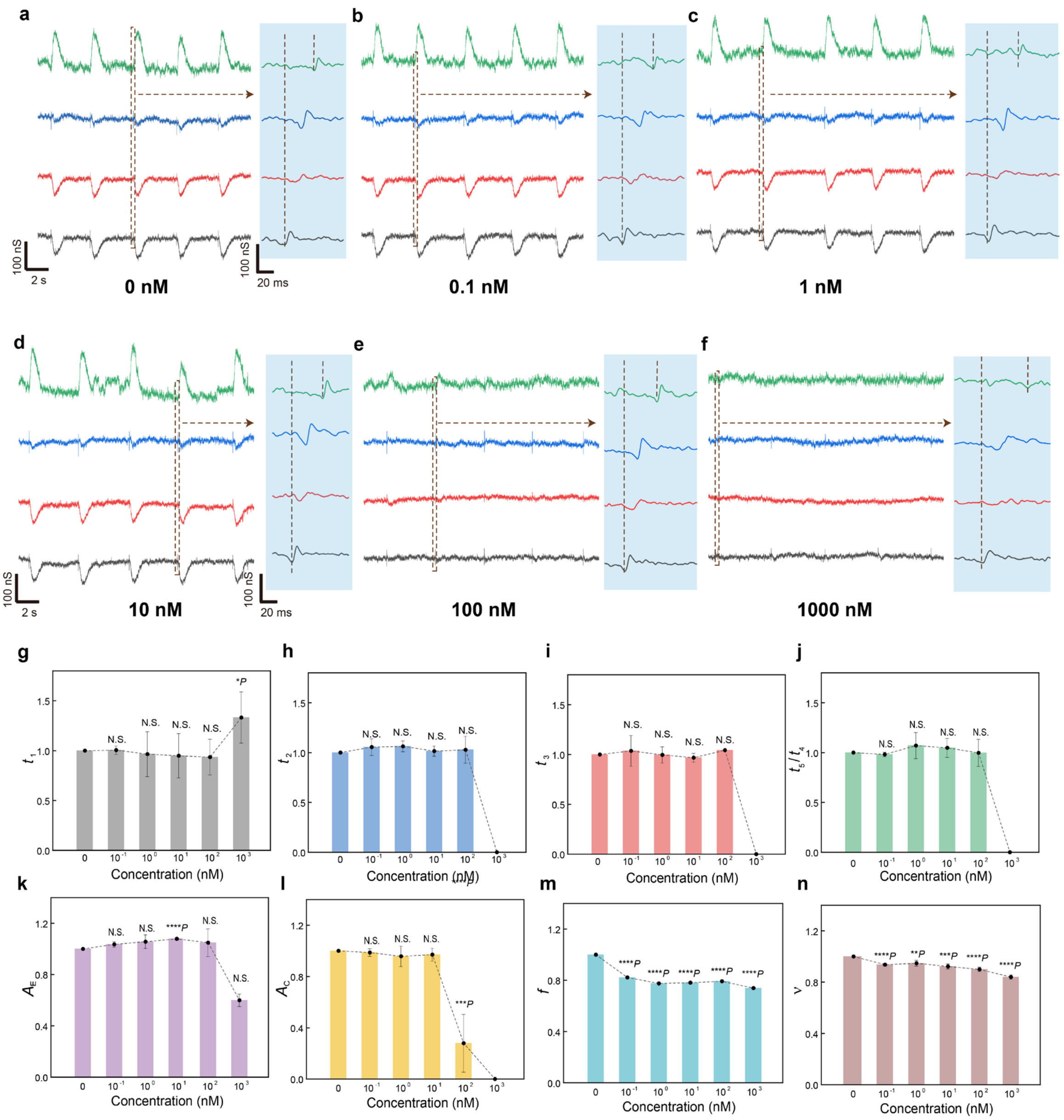

Fig. S23. Dose-dependent verapamil effect on CMT. a-f, Recordings from the same CMT after treatment of verapamil of different concentrations (0 nM to 1,000 nM). The right panel shows the zoom-in action-potential signals from the dash box in each recording. **g-n**, Statistical summary of the extracted parameters (defined in Fig. 3a in the main paper) from the recording signals (n=4 independent devices). All values are normalized to the initial value before verapamil treatment. The results show that verapamil began to introduce obvious inhibition to CMT mechanical response for dosage level ≥ 100 nM. Higher concentration (1 μM) also affected the action potential. Data are presented as mean values ± SD. *P < 0.05, **P < 0.01, ***P < 0.001, ****P < 0.0001, N.S. not significant, using one-way ANOVA with the concentration = 0 nM group as control.

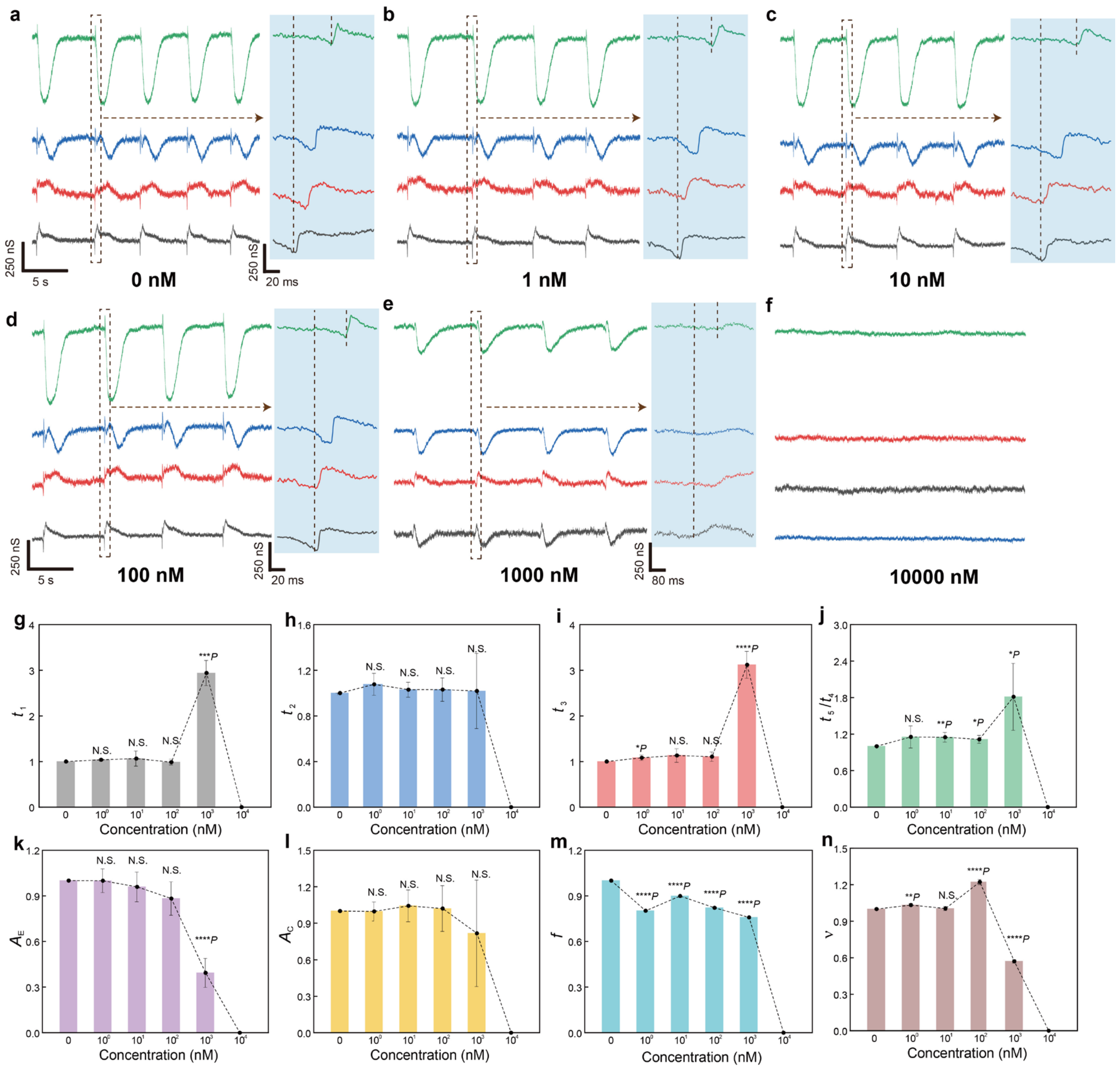

Fig. S24. Dose-dependent quinidine effect on CMT. a-f, Recordings from the same CMT after treatment of quinidine of different concentrations (0 nM to 10,000 nM). The right panel shows the zoom-in action-potential signals from the dash box in each recording. **g-n**, Statistical summary of the extracted parameters (defined in Fig. 3a in the main paper) from the recording signals (*n*=4 independent devices). All values are normalized to the initial value before quinidine treatment. The results indicate that quinidine not only affected the action potential (decreased amplitude and prolongated duration), but also suppressed the mechanical amplitude. Higher concentration (10 μM) fully suppressed both the electrical and mechanical activities. Data are presented as mean values ± SD. *$P < 0.05$, **$P < 0.01$, ***$P < 0.001$, ****$P < 0.0001$, N.S. not significant, using one-way ANOVA with the concentration = 0 nM group as control.

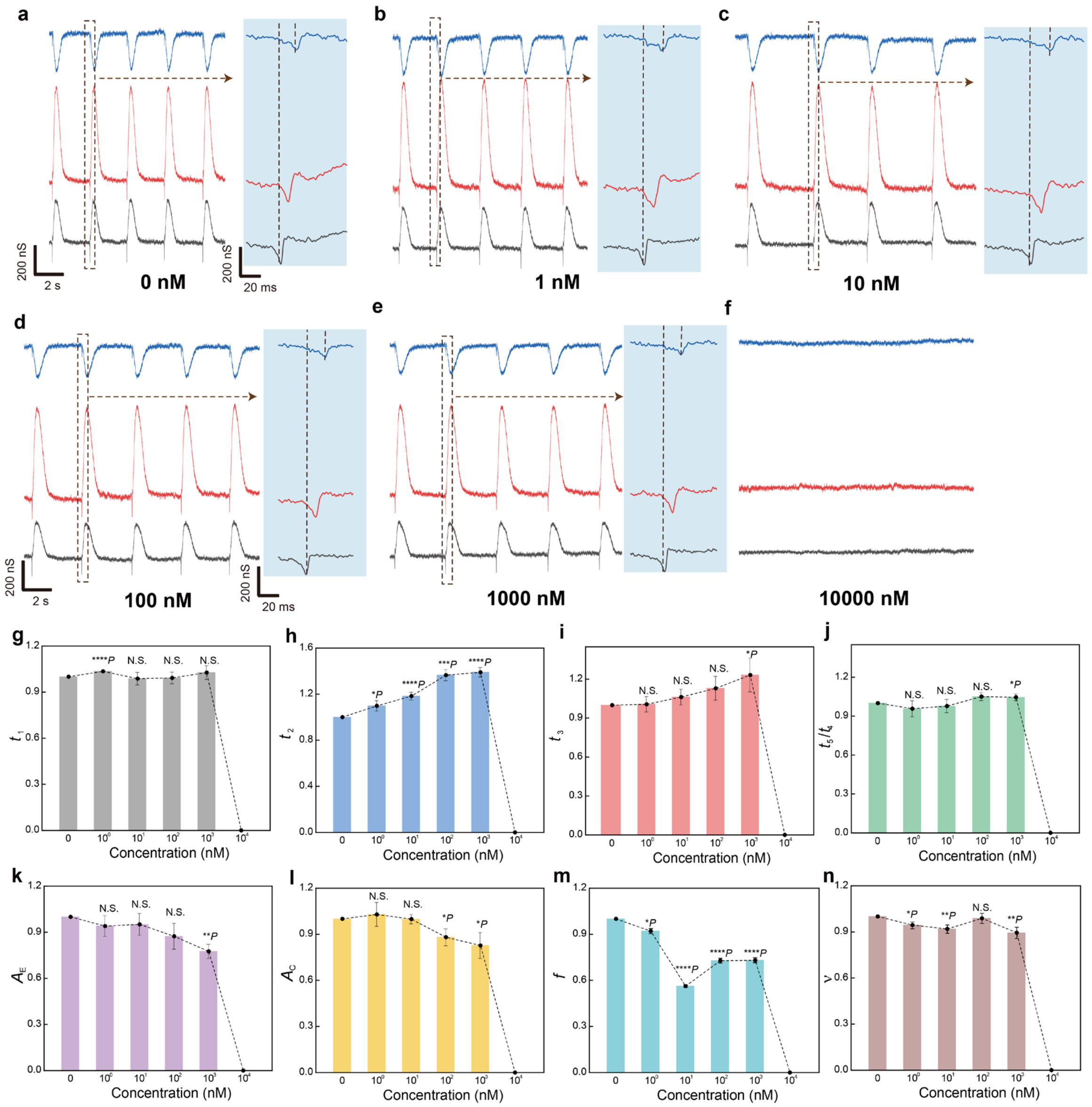

Fig. S25. Dose-dependent doxorubicin effect on CMT. a-f, Recordings from the same CMT after treatment of doxorubicin of different concentrations (0 nM to 10,000 nM). The right panel shows the zoom-in action-potential signals from the dash box in each recording. **g-n**, Statistical summary of the extracted parameters (defined in Fig. 3a in the main paper) from the recording signals (n=3 independent devices). All values are normalized to the initial value before doxorubicin treatment. The results indicate that doxorubicin did not introduce significant effect on the electrical and mechanical functions at low concentration. However, a high concentration (10 μM) could inhibit both electrical and mechanical activities. Data are presented as mean values ± SD. *P < 0.05, **P < 0.01, ***P < 0.001, ****P < 0.0001, N.S. not significant, using one-way ANOVA with the concentration = 0 nM group as control.

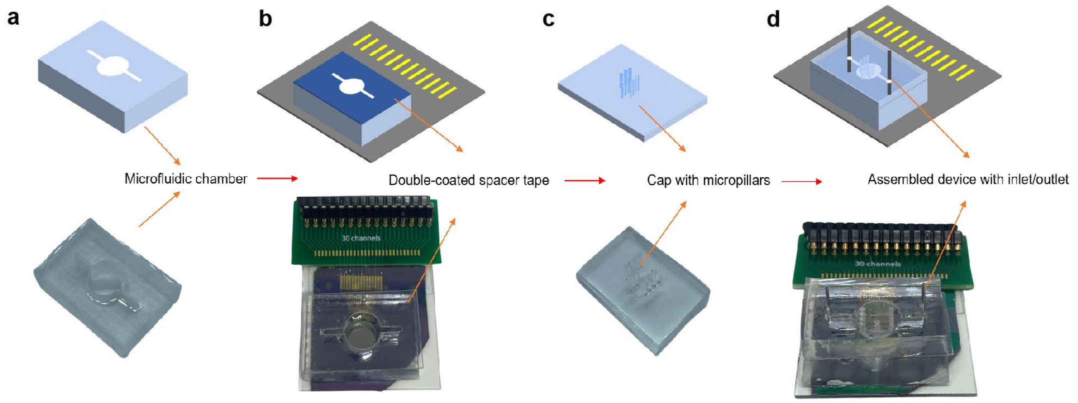

Fig. S26. Assembly of microfluidic chamber for mesh-innervated CMT. **a**, The microfluidic chamber template was created by laser cutting a poly(methyl methacrylate) (PMMA) plate. The dimensions of the middle chamber are as follows: radius: 4.5 mm, height: 7 mm, volume: ~445 μL. Next, polydimethylsiloxane (PDMS; base: cure agent = 10:1) was cast on the template and baked at 100 °C for 3 h. The PDMS layer was then peeled off for the next step. **b**, A thin layer of PDMS glue (base: cure agent = 10:3, 2000 rpm for 60 s) was used to bind the molded PDMS microfluidic layer with the device substrate. The assembly was baked at 80 °C for 2 h. A double-coated spacer tape was added on top of the PDMS microfluidic layer (to bind the cap). **c**, A PDMS cap with micropillars featuring different radii (0.5 mm, 0.4 mm, and 0.3 mm) was similarly molded. The micropillars were designed to eliminate bubbles in the chamber.[7] **d**, The PDMS cap was placed on the PDMS microfluidic channel layer, bounded by the double-coated spacer tape. Inlet/outlet holes were punched and connected with stainless tubes for culture-medium flow.

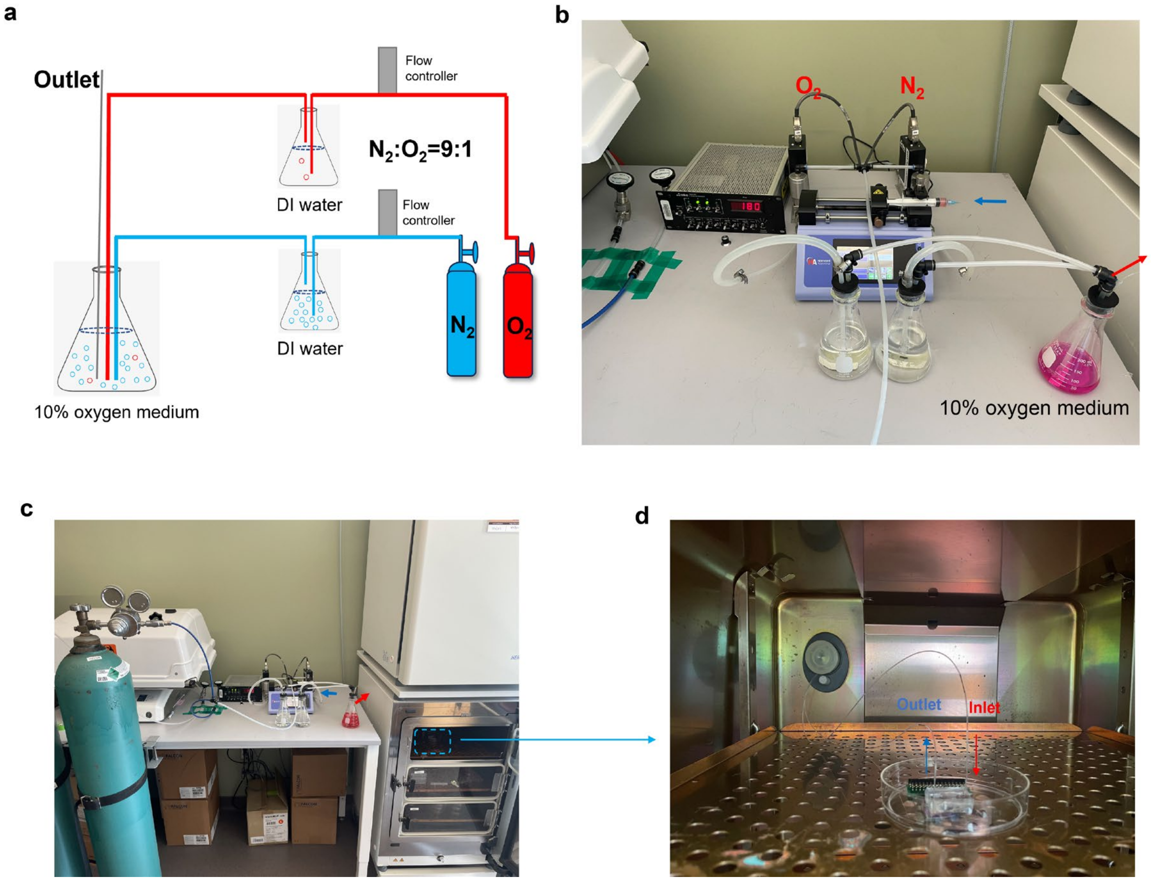

Fig. S27. Oxygen control. a, Schematic of the strategy to generate hypoxic medium flow. The hypoxic medium was prepared by bubbling ultrapure nitrogen and oxygen saturated with water into the culture medium. The gas flow was controlled by a flow controller, and the oxygen concentration was calibrated using an oxygen sensor. The hypoxic medium was injected into the microfluidic chamber using a syringe pump. **b**, The actual flow system. **c**, The entire setup including gas supplies. **d**, The microfluidic system (containing mesh-innervated CMT) in an incubator.

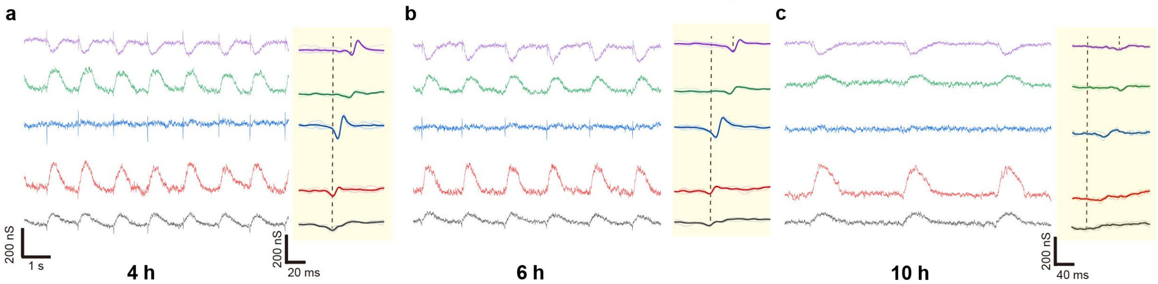

Fig. S28. Electrical recordings from a CMT at different stages of hypoxia. a-c, Recordings after 4-, 6-, and 10-h hypoxic treatment. The right panel in each figure shows the zoom-in superimposed action-potential signals.

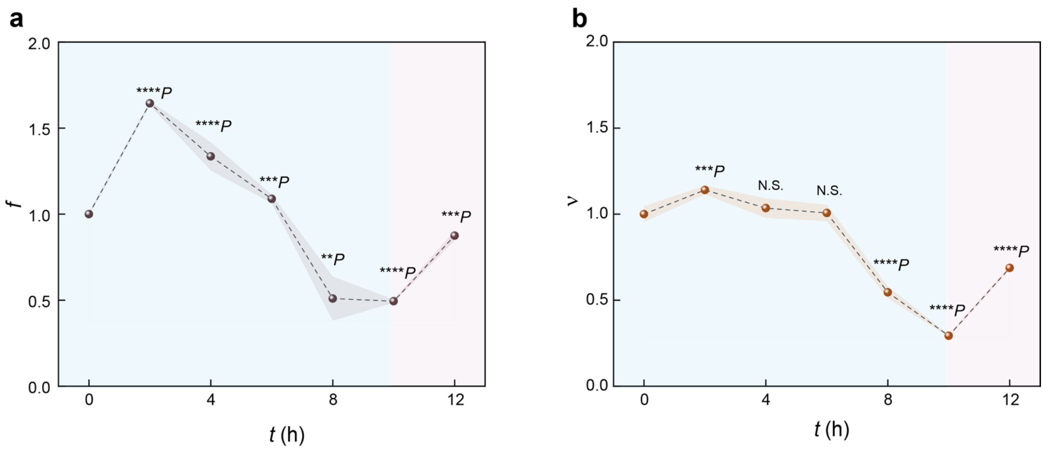

Fig. S29. Evolution of beating frequency and conduction velocity during hypoxia (0-10 h) and normoxia (2 h after hypoxia). Statistical summary of (**a**) beating frequency and (**b**) conduction velocity recorded from the mesh-innervated CMT under hypoxia. All values are normalized to the initial value at 0 h (*n*=5 independent devices). Data are presented as mean values ± SD. $**P < 0.01$, $***P < 0.001$, $****P < 0.0001$, N.S. not significant, using one-way ANOVA with the $t = 0$ h group as control.

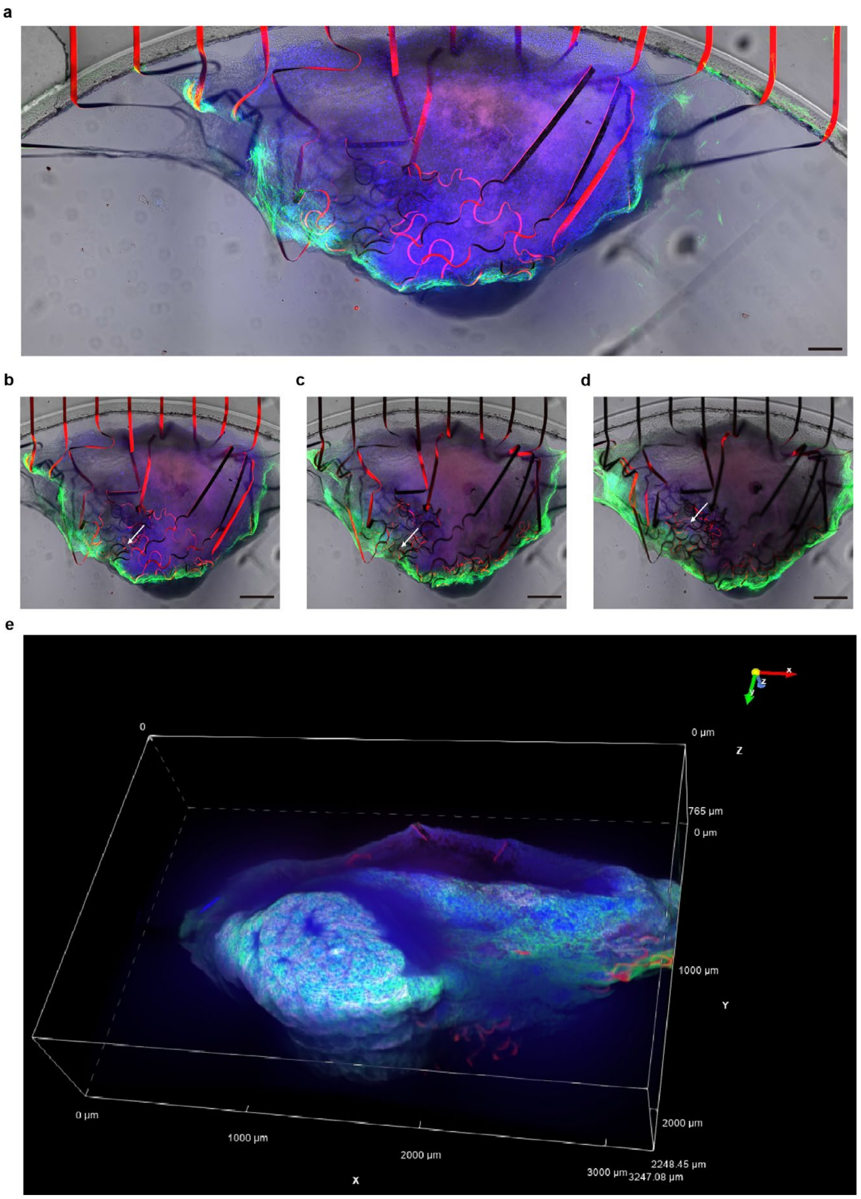

Fig. S30. Imaging mesh-innervated CMT. a, Fluorescence image of the mesh-innervated CMT after disease modeling (overlapped with a bright field image). The red, green, blue, and purple colors indicate mesh, F-actin, 4′,6-diamidino-2-phenylindole (DAPI) and cardiac Troponin T (cTnT), respectively. Scale bar, 200 µm. **b-d**, Fluorescence images showing the positions of three graphene sensor devices (indicated by white arrows) in the CMT. Based on the imaging steps of each device, we can also determine the depth of them within the tissue (~60 µm, 100 µm, and ~150 µm for channel 1, channel 2, and channel 3, respectively). Scale bars, 400 µm. **e**, Reconstructed 3D fluorescence image of the CMT (top-side).

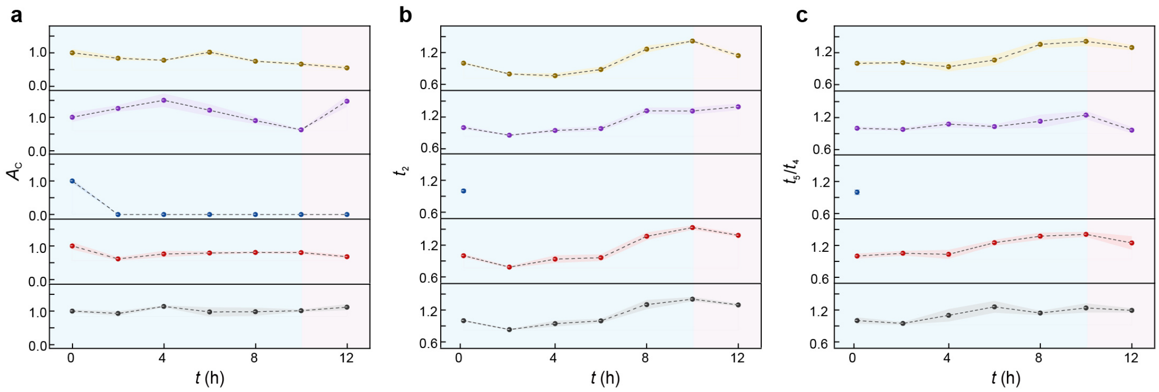

Fig. S31. Evolution of mechanical signal features during hypoxia (0-10 h) and normoxia (2 h after hypoxia). Statistical summary of A_C (**a**), t_2 (**b**), and t_5/t_4 (**c**) in signals recorded from the CMT under hypoxia and normoxia (12 h). All values are normalized to the initial value at 0 h (n=5 independent devices).

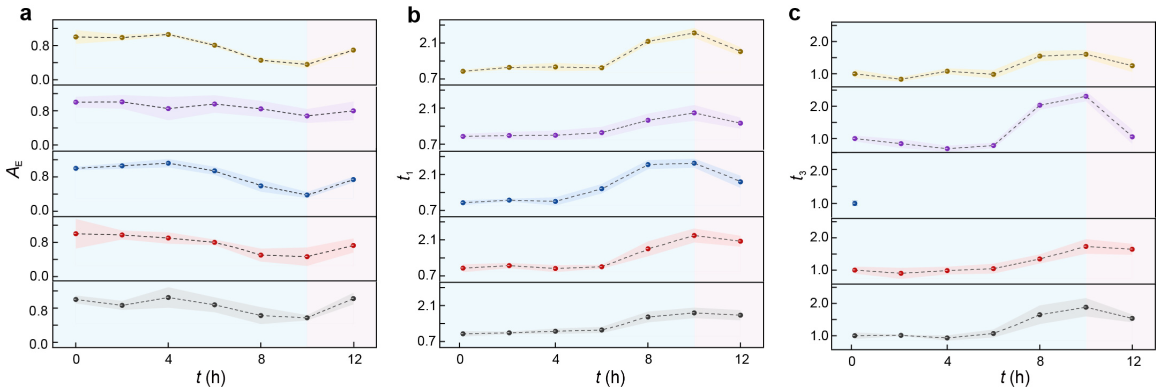

Fig. S32. Evolution of action-potential features during hypoxia (0-10 h) and normoxia (2 h after hypoxia). Statistical summary of A_E (**a**), t_1 (**b**), and t_3 (**c**) in signals recorded from the CMT under hypoxia and normoxia (12 h). All values are normalized to the initial value at 0 h (n=5 independent devices).

Supplementary References

1. Wu, W. et al. Wafer-scale synthesis of graphene by chemical vapor deposition and its application in hydrogen sensing. *Sens Actuators B Chem* 150, 296-300 (2010).
2. Yang, S., Chen, Y. & Jiang, C. Strain engineering of two-dimensional materials: methods, properties, and applications. *InfoMat* **3**, 397-420 (2021).
3. Hess, L.H. et al. Graphene transistor arrays for recording action potentials from electrogenic cells. *Adv Mater* **23**, 5045-5049 (2011).
4. Cohen-Karni, T., Qing, Q., Li, Q., Fang, Y. & Lieber, C.M. Graphene and nanowire transistors for cellular interfaces and electrical recording. *Nano lett* **10**, 1098-1102 (2010).
5. Wang, L. et al. Global strain-induced scalar potential in graphene devices. *Commun Phys* **4**, 147 (2021).
6. Bonaccini Calia, A. et al. Full-bandwidth electrophysiology of seizures and epileptiform activity enabled by flexible graphene microtransistor depth neural probes. *Nat Nanotechnol* **17**, 301-309 (2022).
7. Zhang, Y.S. et al. Multisensor-integrated organs-on-chips platform for automated and continual in situ monitoring of organoid behaviors. *Proc Natl Acad Sci USA* **114**, E2293-E2302 (2017).
