# Supplementary material for: Graphene-integrated mesh electronics with converged multifunctionality for tracking multimodal excitation-contraction dynamics in cardiac microtissues
Source: Nat Commun. 2024 Mar 14;15:2321. doi: 10.1038/s41467-024-46636-7 (PMC10940632; doi:10.1038/s41467-024-46636-7)
Supplement: Supplementary file 3 — Description of Additional Supplementary Files [file 41467_2024_46636_MOESM3_ESM.pdf]

Description of Additional Supplementary Files

File name: Supplementary Movie S1

Description: Synchronized contraction of cardiac microtissue cultured with the mesh electronic system.
