# Supplementary material for: Graphene-integrated mesh electronics with converged multifunctionality for tracking multimodal excitation-contraction dynamics in cardiac microtissues
Source: Nat Commun. 2024 Mar 14;15:2321. doi: 10.1038/s41467-024-46636-7 (PMC10940632; doi:10.1038/s41467-024-46636-7)
Supplement: Supplementary file 5 — Reporting Summary [file 41467_2024_46636_MOESM5_ESM.pdf]

nature portfolio

Corresponding author(s): Jun Yao
Last updated by author(s): 2024-2-12

Reporting Summary

Nature Portfolio wishes to improve the reproducibility of the work that we publish. This form provides structure for consistency and transparency in reporting. For further information on Nature Portfolio policies, see our Editorial Policies and the Editorial Policy Checklist.

Statistics

For all statistical analyses, confirm that the following items are present in the figure legend, table legend, main text, or Methods section.

n/a	Confirmed	
☐	☒	The exact sample size (*n*) for each experimental group/condition, given as a discrete number and unit of measurement
☐	☒	A statement on whether measurements were taken from distinct samples or whether the same sample was measured repeatedly
☐	☒	The statistical test(s) used AND whether they are one- or two-sided<br>*Only common tests should be described solely by name; describe more complex techniques in the Methods section.*
☒	☐	A description of all covariates tested
☐	☒	A description of any assumptions or corrections, such as tests of normality and adjustment for multiple comparisons
☐	☒	A full description of the statistical parameters including central tendency (e.g. means) or other basic estimates (e.g. regression coefficient) AND variation (e.g. standard deviation) or associated estimates of uncertainty (e.g. confidence intervals)
☐	☒	For null hypothesis testing, the test statistic (e.g. *F*, *t*, *r*) with confidence intervals, effect sizes, degrees of freedom and *P* value noted<br>*Give P values as exact values whenever suitable.*
☒	☐	For Bayesian analysis, information on the choice of priors and Markov chain Monte Carlo settings
☒	☐	For hierarchical and complex designs, identification of the appropriate level for tests and full reporting of outcomes
☒	☐	Estimates of effect sizes (e.g. Cohen's *d*, Pearson's *r*), indicating how they were calculated

Our web collection on statistics for biologists contains articles on many of the points above.

Software and code

Policy information about availability of computer code

Data collection	Commercial software pClamp 10.7 was used for electrical data recording and collection. Commerical NIS-Elements (version 5.3) associated with Nikon A1R confocal microscope was used for optical data acquisition.
Data analysis	OriginPro (version 2022, OriginLab);ImageJ (version 2.1.0); NIS-Elements (version 5.3); Microsoft Excel (version 2021); and pClamp 10.7 were used for data analysis.

For manuscripts utilizing custom algorithms or software that are central to the research but not yet described in published literature, software must be made available to editors and reviewers. We strongly encourage code deposition in a community repository (e.g. GitHub). See the Nature Portfolio guidelines for submitting code & software for further information.

Data

Policy information about availability of data

All manuscripts must include a data availability statement. This statement should provide the following information, where applicable:

- Accession codes, unique identifiers, or web links for publicly available datasets
- A description of any restrictions on data availability
- For clinical datasets or third party data, please ensure that the statement adheres to our policy

The data supporting the findings of this study are available within the article and its supplementary files. Any additional requests for information can be directed to, and will be fulfilled by, the corresponding author. Source data are provided with this paper.

Research involving human participants, their data, or biological material

Policy information about studies with human participants or human data. See also policy information about sex, gender (identity/presentation), and sexual orientation and race, ethnicity and racism.

Reporting on sex and gender	N/A
Reporting on race, ethnicity, or other socially relevant groupings	N/A
Population characteristics	N/A
Recruitment	N/A
Ethics oversight	N/A

Note that full information on the approval of the study protocol must also be provided in the manuscript.

Field-specific reporting

Please select the one below that is the best fit for your research. If you are not sure, read the appropriate sections before making your selection.

☒ Life sciences ☐ Behavioural & social sciences ☐ Ecological, evolutionary & environmental sciences

For a reference copy of the document with all sections, see nature.com/documents/nr-reporting-summary-flat.pdf

Life sciences study design

All studies must disclose on these points even when the disclosure is negative.

Sample size	For the in vitro studies, we chose a sample size of n≥3 independent samples per study group for the cardiac microtissue recordings (such as the long-term recording, drug tests, and disease modeling). These sample sizes were sufficient to investigate the efficacy of the treatment as compared to control group with significant difference.
Data exclusions	No data were excluded
Replication	All tests were replicated at least 3 times and performed independently. All attempts at replication were successful.
Randomization	All studies were performed with randomly allocated experimental groups.
Blinding	Data acquisition and analysis were performed by investigators blinded to the groups

Reporting for specific materials, systems and methods

We require information from authors about some types of materials, experimental systems and methods used in many studies. Here, indicate whether each material, system or method listed is relevant to your study. If you are not sure if a list item applies to your research, read the appropriate section before selecting a response.

Materials & experimental systems

n/a	Involved in the study	
☐	☒	Antibodies
☐	☒	Eukaryotic cell lines
☒	☐	Palaeontology and archaeology
☒	☐	Animals and other organisms
☒	☐	Clinical data
☒	☐	Dual use research of concern
☒	☐	Plants

Methods

n/a	Involved in the study	
☒	☐	ChIP-seq
☒	☐	Flow cytometry
☒	☐	MRI-based neuroimaging

Antibodies

Antibodies used	(1) Cardiac Troponin T Monoclonal Antibody (clone number 13-11); 1:250; Invitrogen; Catalog # MA5-12960; LOT 2504985.<br>(2) Alexa Fluor™ 647, Goat anti-Mouse IgG (H+L) Cross-Adsorbed Secondary Antibody; 1:500; Invitrogen; Catalog # A-21235LOT 1987304.

Validation	All antibodies are commercially available and have been tested or validated by the manufacturers (as in their webpages below).<br>Cardiac Troponin T Monoclonal Antibody (13-11): https://www.thermofisher.com/antibody/primary/target/cardiac%20troponin%20t.<br>Alexa Fluor™ 647, Goat anti-Mouse IgG (H+L) Cross-Adsorbed Secondary Antibody: https://www.thermofisher.com/antibody/product/Goat-anti-Mouse-IgG-H-L-Cross-Adsorbed-Secondary-Antibody-Polyclonal/A-21235

Eukaryotic cell lines

Policy information about cell lines and Sex and Gender in Research

Cell line source(s)	hESCs cell line; H9; WAe009-A; WiCell; NIH registration no. 0062; female
Authentication	The cell line is commercially available and have been tested or authenticated by the manufacturers on the basis of standard techniques including morphology check, isoenzyme analysis and mycoplasma detection (as in their webpages below).<br>https://www.wicell.org/home/stem-cells/catalog-of-stem-cell-lines/wa09.cmsx
Mycoplasma contamination	Mycoplasma testing were performed routinely to ensure genomic integrity.
Commonly misidentified lines<br>(See ICLAC register)	No commonly misidentified cell line was used in this study.

Plants

Seed stocks	N/A
Novel plant genotypes	N/A
Authentication	N/A
